# Medical Letter-Bag

**Published:** 1919-06

**Authors:** 


### THE MEDICAL LETTER-BAG

A Department for Short Correspondence from Physicians

"Piece out our imperfections with your thoughts."--Shakespeare

### THE SUCCESSFUL TREATMENT OF GALL-STONE WITHOUT THE KNIFE.

In 1903 I was called to see a woman, Mrs. Charles B., who had called in two doctors who did not understand her case. As I entered the door, a priest was coming out, who had just given her the last rites of the Catholic Church. Truly the poor woman was moribund and deadly still except for convulsive moments that gave the only indication of life. I made an immediate diagnosis and injected 1/3 gr. of morphine and 1/100 gr. of scopolarium right down into the gall bladder as I could feel the lump made by the gall-stones. The convulsions immediately ceased; I then manipulated the gall bladder so as to shove the stones out into the bowel. A few hours later I gave the patient a full dose of calomel and soda with podophyllin. Two days later she began the passage of the gall-stones; they sounded in the bed-pan like the rattle of shot or hail as she ejected them.

Through the sensational reports of the case I was called into another case (it had been diagnosed as everything but gall-stones). This lady was well at the end of two weeks. In her case, although she had ulcer of the gall-bladder from erosion, I managed to pass out the stones by massage. The stones appeared in the stools surrounded by tenacious mucous impregnated with pus and blood. Her agony was intense owing to the ulceration and she was the only patient I ever treated who felt the manipulations even though under the influence of morphine and scopolanine.

A year after this I was called into a very sensational case. A Mrs. Morris, who had been suffering for a year and a half and who had been treated by a dozen doctors with no relief, hearing of me sent for me. I came on Friday evening; the patient was in great agony, nauseated and jaundiced with a temperature of 104° F. I immediately set to work giving her the injection and massage. Relief was almost instantaneous. Besides putting the patient on calomel and soda, etc., to increase the flow of bile, I also used bile salts of F. E. chionanthus. On Sunday morning, Mrs. Morris passed the first stone as big as the end of her thumb; on

Tuesday morning she passed the second and brought it to my office; the two stones together were the size of her thumb.

The sensationalism of this case brought me many others, two of which I shall mention:

Mrs. O. had suffered for two years. In her case the gall-stones could be felt even through much adipose. They felt like pieces of broken glass. After the third attack I pushed them out. The two stones were faceted and my being able to reach them so well had caused the jagged points to be chipped off allowing of easier passage.

Mrs. B. was cured after the third attack also. In her case the stones were very jagged and also faceted from my manipulations. This was four years ago. This lady died recently from pneumonia.

This treatment is simple but wonderful, inasmuch as from the first injection of the scopolaine and morphine near or into the gall-bladder the character of the suffering changes and the patient never undergoes the same agony.

I have cured in all about twenty patients and only had to turn one over for operation on account of complications.

DR. G. C. BOUDOUSQUIE,
New Orleans, La.

---

**Translation.**

Nature smiled
Thru early morning mists.
Soft moonbeams, palm-reflected,
Retreating before solic rays,
The two commingling in rainbows splendor caught
Of dew drop crystal.
And morn awakening
Kissed with the thorofied draught
The Rosebud,
Which straightway lifted
A smiling face.

Salvadore Corone's black Nanny goat,
Sans aestheticism, ate the rose to
Satisfy her hunger crave,
Showing her goat joy.
So Nature's smile traveled on.

Black Nanny's milk fed

Dainty Cossette,
Dark Italian Kiddie;
Sustained her romping feet
Through golden sunshine hours.
In her crib at evenfall
Cossette, her bottle empty,
Smiled.
A lone star-beam
Caught that baby-gleam
And back to Nature it returned.

There is truly an "all-sustaining thread" which "runs thru all and doth all unite." The same science which weighs accurately the unthinkable bulk of distant suns weighs exactly the electron which no conceivable power can render visible to the eye. And science has discovered the One Law from electron to sun.

All life is rhythm and harmony; death is but maladjustment. The mystic scale of seven repeats in music in an ascending rate of vibrations. In like manner the mystic spectrum of seven colors. In the Kratone Laboratories recently I gazed thru a little instrument, the invention of my close friend, Prof. W. Scott Lewis, and saw the ultraviolet rays of red on higher scale than mortal eye has ever discerned except in this one laboratory.

Just a wee bit of transmutation. The search for the philosopher's touchstone thru alchemy brot the miracle of chemistry and modern chemistry has learned what it ridiculed in the alchemist —that matter and energy are one. Astrology led to astronomy, but a higher astronomical knowledge had preceded astrology; and modern astronomy is finding the truths of stellar influence it once derided. The materialist mocked the metaphysician; then by mutual studies they learned that both were but partly right and their parti-truths blended into the new mysticism.

No physician claims to "cure" (unless he is very young). You realize, of course, doctor, that Nature does the curing. You are but the means of helping Nature to get a chance. In the past our healers sometimes got in the way of Nature, but we are learning now that there are unseen forces working in the marvelous laboratory and factory of the body and that we must learn more and more to "keep off the soul" as well as to "keep off the grass" in the park.

I met a big, barking dog today, all bristled up and disputing my right to the bypath. I just smiled at him—my brother—and he responded at once. Without a word—simply by the power of good fellowship and sincere love which I held for him—he wagged his tail and the bristling becomes smoothed. We smile together I talked softly to him and in his soul language he spoke to me. It was the transmutation of Nature's smile.

Love is the doctor's biggest asset. There is a physical body to deal with, but it is only the outer casing of the inner ego. Try the transmutation-effect of a smile. I know you do, doctor! And doesn't it work famously?

GUY BOGART,
Los Angeles, Calif.

---

**Pharmacy Correspondence Institution.**

Orange Grove, Texas, June 6th, 1919.

Texas Medical Journal:

Please let me know where I can obtain a course in Pharmacy through correspondence.

Yours truly,
PERRY KLATT.

(Will some one furnish him the desired information?—Ed.)

---

### BOOK REVIEW.

ESSENTIALS OF SURGERY. *A Textbook of Surgery for Students and Graduate Nurses and for those Interested in the Care of theSick.* By Archibald Leete McDonald, M. D., Johns Hopkins University. Formerly in charge of the Department of Anatomy, University of North Dakota; Lecturer on Surgery, Nurses Training School, St. Luke's Hospital, Duluth, Minnesota. 46 Illustrations. Philadelphia and London: J. B. Lippincott Company, 1919. Price: $2.00 net.

Within the bounds of a little over 200 pages, this little manual on surgery presents the vast subject of surgery in a condensed, yet simple manner. It easily may be said that most of the essentials of surgery are touched upon. Of necessity, mere mention of most diseases and their prominent features is made. But for an intelligent, comprehensive, and well proportioned introduction to the extensive field of surgery, this book may be recommended without hesitation to those interested in the care of the sick. A glossary at the end of the book will greatly facilitate an understanding of the subject as treated, for it contains definitions of the technical terms unavoidably used in the book's preparation. A. C. GARCIA.

